# Open Science for private Interests? How the Logic of Open Science Contributes to the Commercialization of Research

**DOI:** 10.3389/frma.2020.588331

**Published:** 2020-11-10

**Authors:** Manuela Fernández Pinto

**Affiliations:** Department of Philosophy, Center of Applied Ethics, Universidad de los Andes, Bogotá, Colombia

**Keywords:** commercialization of science, open Science, open access, industry-funded research, democratization of science

## Abstract

Financial conflicts of interest, several cases of scientific fraud, and research limitations from strong intellectual property laws have all led to questioning the epistemic and social justice appropriateness of industry-funded research. At first sight, the ideal of Open Science, which promotes transparency, sharing, collaboration, and accountability, seems to target precisely the type of limitations uncovered in commercially-driven research. The Open Science movement, however, has primarily focused on publicly funded research, has actively encouraged liaisons with the private sector, and has also created new strategies for commercializing science. As a consequence, I argue that Open Science ends up contributing to the commercialization of science, instead of overcoming its limitations. I use the examples of research publications and citizen science to illustrate this point. Accordingly, the asymmetry between private and public science, present in the current plea to open science, ends up compromising the values of transparency, democracy, and accountability.

## Introduction

Science studies scholars, including a number of historians and philosophers of science, have raised important concerns regarding the current trend toward the privatization and commercialization of scientific research. Financial conflicts of interest, several cases of scientific fraud, and research limitations from strong intellectual property (IP) laws have all led to questioning the epistemic and social justice appropriateness of industry-funded research. At first sight, the ideal of Open Science, which promotes transparency, sharing, collaboration, and accountability, seems to target precisely the type of limitations uncovered in commercially-driven research.

Despite these laudable goals, the plea to open science has primarily focused on publicly funded research. In this paper, I argue that this particular focus challenges the appropriateness of the Open Science movement. As the philosophical analysis of this paper shows, if advocates of Open Science promote the openness of publicly funded research to foster, at least in part, new business opportunities and joint private-public ventures, as well as new markets for the development of online information and communication technologies (ICTs), then Open Science ends up contributing to furthering the commercialization of science, without addressing any of the epistemological and social justice concerns that have been identified. Accordingly, the asymmetry between private and public science, present in the current plea to open science, ends up compromising, not promoting, the values of transparency, democracy, and accountability. In other words, insofar as Open Science fails to acknowledge, analyze and evaluate the structural connections between public science and private interests, it also fails to fulfill its goal of making scientific practices more transparent, democratic and accountable.

In order to show this, the paper is divided into four sections. The first section explains the epistemological and social justice limitations of industry-funded research. The second section introduces Open Science as an ideal that, at first glance, has the potential of overcoming some of the limitations highlighted in the first section. The third section problematize this claim, showing that Open Science, at least in the way it has been implemented, contributes at least in three different ways to the goals of industry-funded science. Finally, the fourth section illustrates how this has happened in two aspects of the scientific process: research publications and citizen science projects.

## Concerns Regarding Privately Funded Science

Over the past decades, scientific research has undeniably moved into the private sector to the extent that nowadays the majority of scientific research is both conducted and funded by the private industry (Eurostat, 2018; National Science Board 2018). As a reaction, a number of scholars have expressed concern about this current trend (Greenberg, 2001; Bok, 2003; Slaughter and Rhoades, 2004; Wise, 2006; Greenberg, 2007; Resnik, 2007; Radder, 2010; Holman and Elliott, 2018). Their main fear is that industry-funded research might have a negative influence on scientists, who, attracted by generous funding schemes, might compromise, perhaps irreparably, the quality of scientific research (Wise, 2006: 1266). The commercialization of science is thus taken as a major threat to scientific rationality, as it possibly puts in jeopardy the normative standards of the scientific enterprise.

Following a number of red flags, industry-funded science has come under increasing scrutiny. The first and most salient warning sign is perhaps the number of scandals in scientific research tied to corporate sponsorship, including the tobacco industry’s cover-up of the health hazards of smoking, the petro-chemical industry’s support of climate skeptics, and the pharmaceutical industry’s manipulation of data in clinical trials, such as in the Vioxx case, among many others (Markowitz and Rosner, 2002; Sismondo, 2007; McGarity and Wagner, 2008; Michaels, 2008; Oreskes and Conway, 2010; Proctor, 2011).

A second red flag, and even more significant from a scientific point of view, is related to results from randomized controlled trials and systematic reviews showing that financial conflicts of interest have a statistically significant effect on research results (Bekelman et al., 2003; Lexchin et al., 2003; Sismondo, 2008; Lundh et al., 2017), in which design bias and publication bias seem to play an important role (Smith, 2005; Doucet and Sismondo, 2008). This empirical evidence suggests that the private funding of scientific research can have an almost imperceptible effect on research results in favor of the commercial interests at stake. Moreover, the most recent meta-analyses show that industry bias can impact research results, even when the quality of the study, measured by standardized statistical tools, is not compromised (Lundh et al., 2017). While this evidence focuses on medical research, conflicts of interest have also been identified as deeply problematic for scientific research more generally (Resnick, 2007; Elliott, 2008).

A third red flag is related to intellectual property rights. As some have suggested, and contrary to what’s expected, strong IP rights, including patent law, can actually inhibit scientific research (Biddle, 2014). IP rights give private companies control over industry-funded research, which has led in turn to coverups of research results that would have been both epistemically and socially relevant (see, e.g., Biddle, 2007; Turner et al., 2008), as well as to impeding or restricting research initiatives (see, e.g., Waltz, 2009; Sappington et al., 2010).

As a consequence, industry-funded research faces important epistemic challenges, insofar as we have good reasons (or at least a number of red flags) to question the influence it has on research outcomes. In addition, many of these epistemological shortcomings have a clear societal impact. For instance, scientific fraud, as well as the subtler mechanisms that have been used by the private industry to obstruct the production of knowledge, in cases such as tobacco smoking, drug development, and climate change (Fernández Pinto, 2017), do not only impact the quality of the knowledge produced, but also the people who depend on that knowledge. As a result, the lack of proper knowledge has led to major human and environmental harms. Accordingly, industry-funded research also faces an important social justice challenge to prove that it can be both commercially-driven and socially responsible (Fernández Pinto, 2018).

These concerns become even more salient, given that the vast majority of scientific research today is both funded and performed in the private/business sector, as has been previously acknowledged. According to the most recent National Science Board indicators of 2018, 72% of scientific research and development (R&D) in the US is performed in the business sector, and 67% is funded by the business sector. One can find a very similar trend in the European Union (Eurostat, 2018) and also worldwide (UNESCO, 2015). In the US, 83% of business R&D performance can be accounted for by five sectors, which include chemical manufacturing (pharmaceuticals), and information (including software publishing industry). If the majority of scientific research is both funded and performed in the private sector, and if commercially-driven science faces important challenges, then we should have some concerns about the current organization of scientific research.

## Open Science to the Rescue?

Before examining the potential of Open Science for countering the problems found in commercially driven research, it is important to notice that the concept of open science is not used unequivocally.^1^ Sometimes the concept is used as an ideal to be obtained, a “scientific culture characterized by its openness” (Bartling and Friesike, 2014). In this sense, Open Science is a goal for research, which promotes transparency, accessibility, and collaboration, among other values.^2^ Other times the concept is used to describe a movement within the scientific community, which also promotes certain values, but is mainly in charge of building the structures and designing the policies that would eventually lead to openness, as well as of advocating for the ideal and convincing others of its importance, e.g., “the immediate challenge for the Open Science movement is its ability to change the culture of science itself, which continues to operate within a print-based, proprietary, and closed framework for scientific discovery and communication” (Sidler, 2014: 82). Yet other times the concept is used to describe the set of policies that should be implemented to promote the core value of openness (e.g., Levin et al., 2016). Additional uses might also be at play, such as the idea of open science as a project or as a research strategy, among others. Part of the issue at hand here is related to the fact that there is a disconnect between the ideal of Open Science, and how this ideal is implemented through Open Science policies and promoted by the Open Science movement. As it will become clear in what follows, the argument of this paper does not question the ideal per se, but instead it questions the particular way the ideal has been conceived and implemented by the Open Science movement, as well as the way it has been brought about through Open Science policies. In this sense, the faulty logic of open science that I aim to highlight in the paper refers precisely to the inconsistency between the ideal and its current implementation.

At first sight, the ideal of Open Science, promoting what one might consider traditional democratic values, such as transparency, accessibility, collaboration and accountability, and arguing for a more inclusive, diverse, and pluralistic science, seems to target precisely the type of limitations uncovered in commercially-driven research. After all, these are the values that scientific research done in the private sphere sorely lacks, and that presumably have led to some of the main methodological and social justice issues that have been uncovered. More transparent and open venues for data collection and storage, peer review of methodological decisions and experiment designs, opening the peer-review process for paper publication, and open venues for publications themselves, all seem to point in the right direction for counteracting the state of secrecy and protection that characterizes commercially-driven research today.

In principle, the three main problems highlighted in the first section could benefit from more openness. First, scandals in industry-funded science, such as the tobacco industry’s denial of the health hazards of smoking or the many episodes of data manipulation and undisclosed results from the pharmaceutical industry (e.g., in the cases of Bextra, Celebrex, Fen-Phen, Redux, and Vioxx), would be less encouraged in an environment where the research process and results are submitted to open peer evaluation and accountability (Maurer 2007: 426; Royal Society, 2012: 8). In this sense, initiatives to open up the research process from the early stages, e.g., through peer evaluation of experimental design, as well as strategies to evaluate published research through post-publication open venues, would keep scientific results under surveillance, buttressing the self-regulatory aims of science (Meskus et al., 2018).

Second, the pervasive financial conflicts of interest and its influence on research results, for which we now have strong evidence (Lundh et al., 2017), would also benefit from more transparency. If nothing else, more transparency means at least disclosure of conflicts, the first step toward plausible management strategies (Elliott, 2008). Moreover, the biasing mechanisms that might be in place in cases where industry-funding influences research results without compromising the quality of the studies, which are mostly imperceptible through standard quality assessment tools, might be easier to identify or even completely avoided through Open Science policies. For instance, problems arising from cherry-picking significant outcome measures post hoc (Andrade, 2015), could be countered by open registration of study design and protocols (such as, ClinicalTrials.gov), which includes determining primary outcome measures before the research starts.

Finally, strategies to open research in the strong IP regime we currently live in would encourage more scientific research on patent protected materials, as well as more non-protected data and publications, which in the long run is expected to achieve better and more reliable knowledge (Royal Society, 2012). Additionally, in all three aspects, citizen participation in the form of real interaction between industry and stakeholders regarding the social relevance and benefits of the research pursued would importantly contribute to making commercially-driven research more socially responsible.

Undeniably, the values that inspire the ideal of Open Science are promising guidelines to face the epistemic and social justice challenges of research done in the private sphere. In fact, much of the rhetoric use to promote and encourage Open Science explicitly targets some of the epistemic and social justice problems mentioned before. For instance, according to the OECD (2015) report “Making Open Science a Reality”:

Open search tools increase the efficiency of research as well as of its diffusion. Greater access to scientific inputs and outputs can improve the effectiveness and productivity of the scientific and research system, by: reducing duplication costs in collecting, creating, transferring and reusing data and scientific material; allowing more research from the same data; and multiplying opportunities for domestic and global participation in the research process. Scientific advice can also benefit from the greater scrutiny offered by open science, as it allows a more accurate verification of research results (…) Open science also allows the closer involvement and participation of citizens (OECD, 2015: 10).

In this way, Open Science is presented as a policy strategy to achieve better and more efficient scientific knowledge, as well as closer citizen participation in the scientific process.

Openness is also frequently portrayed as a core value of modern science, with an almost mandatory appeal to Robert Merton’s ethos of science (Schroeder, 2007). According to Merton’s rule of communism or communalism, “The substantive findings of science are a product of social collaboration and are assigned to the community,” making secrecy “the antithesis of this norm” (Merton, 1974: 271). The ideal of Open Science is thus aligned with the traditional scientific ethos, only to be further supported by ICT revolution. In other words, Open Science policies in the 21st century would instantiate the scientific value of communalism granting access through different types of ICTs, such as open access journals (e.g., PLOS), open electronic archives (e.g., arXiv), collective intelligence projects (e.g., Polymath), public computing projects (e.g., Rosetta@home), citizen science projects (e.g., the Galaxy Zoo Project), collaborative research environments (e.g., Open Science Grid), academic social networks (e.g., ResearchGate and academica.edu), and social reference managers (e.g., Mendeley and Zotero), among others.

## Open Science as a Business Strategy

Despite the endorsement of the ethos of science and the promising strategies to open science through ICTs, I argue that Open Science, at least in the way it has been implemented, contributes at least in three different ways to the goals of industry-funded science. First, Open Science has been initially conceived only for publicly funded science, leaving it open for the private industry to join or not at its convenience. Second, Open Science has also been conceived to respond to the demands of the private sector. And third, Open Science also seems to foster a new way of commercializing science through development of new ICTs. Let me explain these in turn.

### Opening Publicly Funded Science

The Open Science movement and its policies target primarily publicly funded research, while remaining silent about the problems already uncovered in commercially-driven science. Evidence for this claim can be found in multiple venues. Michael Nielsen, the author of *Reinventing Discovery: The New Era of Networked Science* (Nielsen, 2011a) and a strong advocate of Open Science, has stated a number of times, including during his TED talk “Open Science now!,” that “any publicly funded science should be open science” (Nielsen, 2011b, 13:14–13:20). In a similar vein, the OECD defines Open Science as “the efforts by researchers, governments, research funding agencies or the scientific community itself to make the primary outputs of *publicly funded research* results—publications and the research data—publicly accessible in digital format with no or minimal restriction as a means for accelerating research” (OECD, 2015: 7, emphasis mine). Perhaps even more explicitly, in 2013 the G8 Science Ministers agreed on the following statement: “To the greatest extent and with the fewest constraints possible *publicly funded scientific research data should be open*, while at the same time respecting concerns in relation to privacy, safety, security and commercial interests, while acknowledging the legitimate concerns of private partners” (G8 Summit, 2013, emphasis mine), making clear that their support of open science was limited to publicly funded research and respectful of agreements with the private sector.

A comparable view is commonly turned into justification for granting open access to scientific publications, arguing that “research funded by tax-payers should be made available to the public free of charge so that the tax-payer does not in effect pay twice for the research—first for the research to be done and then to read the results” (Phelps et al., 2012: 1). Given that tax-payers pay for publicly-funded research, they have a right to access the results of such research. However, as the reader can see, the argument only applies to publicly-funded research, leaving all forms of privately-funded and privately-performed research outside the scope of Open Access. The recently approved Guidelines on Open Access to Scientific Publications and Research Data in Horizon, 2020, according to which the EU endorses the open access of scientific publications and research data, states that “under Horizon, 2020, each beneficiary must ensure open access to all peer-reviewed scientific publications relating to its results” (European Commission, 2016: 5). The policy is however mandatory only for beneficiaries of H2020 grants, which again restricts open access to publicly funded research.

In the meantime, private companies remain in the privileged position of adopting openness as they see fit. Pharmaceutical companies, for example, might benefit from Open Data strategies, e.g., developing public data bases, and strengthening international collaborations and networks. Given the amount of time and resources that is required to obtain marketable treatments from raw data, pharmaceutical companies might benefit more from Open Data than from maintaining data confidential. As Leonelli claims:

…many rich laboratories have found that data donation offers the opportunity to participate in international networks and receive help with data analysis, thus accruing their own prestige, visibility, and productivity. Even major pharmaceutical companies like GlaxoSmithKline and Syngenta are contributing to the development of public databases, in the hope of outsourcing their R&D efforts, improving their public image, and gaining from the availability of data produced through public funding (Leonelli, 2013: 9).

Presumably, private companies might be less likely to share data analyses that are unfavorable to the industry, as has happened a number of times with pharmaceutical companies covering up research results that can impact their market sales. A clear example of this is the infamous case of research on antidepressants. According to a study conducted by Turner and colleagues, where they compared the published literature against the studies reported by the US Food and Drug Administration, selective publication of clinical trial on 12 antidepressants resulted in a 32% overestimation of effect size in the published literature (Turner et al., 2008: 255–56). In other words, due to the fact that the industry decided to publish only favorable results, the efficacy of antidepressants was importantly overestimated. As this case shows, the industry has huge market incentives to maintain unfavorable research results in the dark.

In sum, while the ideal of Open Science endorses key values to counteract the epistemic and social shortcomings uncovered in commercially-driven research, the Open Science movement and its policies have primarily focused its efforts in opening publicly funded research, leaving the private industry free to decide about openness as it finds convenient.

### Responding to Business Demands

Limiting Open Science to publicly-funded research should not come as a surprise. After all, it is easier to argue, legally at least, that tax payers have the right to access scientific results obtained through government funding than to make the case for opening research done in the private sector. Accordingly, one might claim that opening publicly funded science is a first and firm step toward a more transparent and accountable scientific process in general. Efforts against Open Science on the contrary would not lead science in the right general direction.

However, one could also argue that Open Science has also been conceived to respond to business demands. In fact, the plea for opening publicly funded science is commonly supported by the possible commercial ventures that opening science might encourage. The OECD and UNESCO both use this argument in favor of Open Access policies: “Scientists and academics are not the only groups that can benefit from greater open science efforts. The demand from the *business sector* and individual citizens to access research results is significant” (OECD, 2015: 11, emphasis mine; see also UNESCO, 2012: 29). In fact, Open Science policies are commonly encouraged as a way to grant access to scientific knowledge not only to other researchers and the public, but also to private industry. The H2020 goals of the EU make exactly this point: “This means making publicly-funded scientific information available online, at no extra cost, to European researchers, *innovative industries* and the public, while ensuring that it is preserved in the long term” (European Commission, 2016: 5, emphasis mine).

The resulting document from the *Open Science—From Vision to Action* EU presidency conference hosted by the Netherlands in April 2016 explicitly states at the outset that Open Science is good for business:

Open science also increases business opportunities. The speed at which innovative products and services are being developed is steadily increasing. Only companies (…), entrepreneurs and innovative young people that have access to the latest scientific knowledge are able to apply this knowledge and to develop new market possibilities (EU Presidency, 2016: 4).

In addition, many advocates also see potential commercial uses as a reason to favor Open Science initiatives. For instance, Open Knowledge International, an international network advocating Open Science, states as one of its four core principles that:

The use of licenses which limit commercial re-use or limit the production of derivative works by excluding use for particular purposes or by specific persons or organizations is STRONGLY discouraged. These licenses make it impossible to effectively integrate and re-purpose datasets and prevent commercial activities that could be used to support data preservation.

If you want your data to be effectively used and added to by others it should be open as defined by the Open Knowledge/Data Definition—in particular non-commercial and other restrictive clauses should not be used.* (Murray-Rust et al., 2010.)

As the principle suggests, restricting commercial uses of data would be counteractive for open data. In sum, major efforts to implement Open Science have focused on publicly funded research, making results from this research widely available, while leaving aside or simply ignoring the lack of openness in the private sector. At the same time, many of these efforts identify potential commercial ventures as a desirable outcome of Open Science. Or in other words, if opening publicly funded research helps support private, commercial, or industrial endeavors, then the more reasons we have to favor Open Science policies.

### Creating New Ways of Commercializing Science

Furthermore, Open Science also seems to foster a new way of commercializing science, for the opening of science in the 21st century does not come in the form of public forums in the agora or through open access to public libraries worldwide. The Open Science movement is very clear in this respect: we ought to take advantage of the unrestricted possibilities that ICTs give us, especially through online platforms, to take open science at a new level—what is also known as Science 2.0. But this means that opening science today comes together with an increasing number of online open access platforms, an “e-infrastructure” as Schroeder (2007) has called it, mostly developed through a Silicon Valley startup model, aiming at the likes of Facebook and Google; i.e., another form of venture capitalism under the rhetoric of democracy and citizen participation.

A new “knowledge industry,” as Fecher and Friesike (2014) have called it, is slowly but surely emerging from implementing open science. One only needs to look at the number of different types of ICTs developing new business models for open science. As Mirowski (2018) has documented, Open Science seems to operate through the new business model of platform capitalism, in which all the contents of the research process, from study design, to data collection, to peer-review, and publication, are expected to be available on online platforms. We are already witnessing how a number of platforms compete to be the go-to online repository of a specific aspect of the research process. Take, for example, academia.edu and ResearchGate competing to be the mandatory Facebook of science.

In sum, Open Science, with its focus on publicly funded science and its encouragement of new ICTs and new commercial ventures, is not merely overlooking commercial research, but actually contributing to strengthen it. While some might consider this an asset of Open Science, given that it seems to promote both transparency and business opportunities, I prefer to be cautious about this win-win reading of the situation. As I mentioned in “*Concerns Regarding Privately Funded Science*” section, commercial interests have had a worrisome influence on the scientific process, moving science away, not toward the ideals of transparency, democracy, and accountability, promoted by open science. In the next section, I examine two further examples to illustrate this point, i.e., the cases of publication planning and of citizen science projects.

## The Problem Illustrated by Two Cases

If the previous analysis is correct, the Open Science movement has an asymmetric view of private and public research, according to which openness has only been applied to publicly funded science; and this asymmetry sets up publicly funded science for further commercial gain. If this is so, Open Science has not really contributed to ameliorating the epistemic and social justice problems in commercially driven research, but instead seems to contribute to them. The question arises whether Open Science is properly aligned with the values of transparency, democracy, and accountability that the movement fiercely promotes, or if it ends up compromising such values. In order to address this question, let us examine two cases of interaction between Open Science and commercial interests: first, the interaction between open access and publication planning, and, second, the interaction between citizen science projects and participatory research.

### Open Access and Publication Planning

Although Open Science is a much broader project than the implementation of open access to publications and data sets, open access is certainly one of the main pillars of Open Science. In order to set the common ground, let’s start with a fairly standard definition of Open Access:

Open Access (OA) is the provision of free access to peer-reviewed, scholarly and research information to all. It requires that the rights holder grants worldwide irrevocable right of access to copy, use, distribute, transmit, and make derivative works in any format for any lawful activities with proper attribution to the original author. Open Access uses Information and Communication Technology (ICT) to increase and enhance the dissemination of scholarship. OA is about Freedom, Flexibility and Fairness (UNESCO, 2012: 6).

The idea of open access is thus closely connected to Merton’s norm of communalism or the idea that scientific knowledge does not belong to anyone in particular, but to all, i.e., to the human community at large. In this sense, every single person has a right, not only to access scientific results, but also to use that knowledge for “any lawful activities.” Through Open Access scientists (or whoever has the IP rights) grant the public the right to use the knowledge produced at no cost.

Open access comes in different flavors, but two of the most common ways to implement it are through the “gold” and “green” models. Gold Open Access works basically like traditional publishing going through the peer-review process, only that authors (or their institutions) do not wave their IP rights to the journals, but instead pay a fee for publication. For example, PLOS, one of the most successful venues in this respect, charges a fee ranging between 1,495 and 2,900 dollars per published article. In contrast, the green model encourages authors to upload their articles in a public repository, which can be done pre-print and without peer-review, or post-print after the traditional peer-review process. Some of the most commonly used venues are, for example, arXiv, ResearchGate, and academia.edu.

Consider now the interaction between these open access practices and a commonly used strategy for successful publishing in the pharmaceutical industry, i.e., publication planning. As Sismondo has documented in detail, the pharmaceutical industry frequently uses publication planning firms to ensure that their articles are published in the best medical journals, reaching the vast majority of doctors who would potentially prescribe their medications. The process is carefully handled from the very early stages. Pharmaceutical companies out-source clinical trials through contract research organizations and publication planning firms make sure that the articles are (ghost)written in industry-friendly ways and that they are signed by “independent” researchers. Sismondo describes the process as follows:

Most sponsored clinical trial research is handled by contract research organizations (CROs), the data they produce is typically analyzed by pharmaceutical company statisticians, papers are written by medical writers, and the whole process is guided and shepherded through to publication by planners and planning teams […]. To gain the most commercial value from research, the papers publicizing it are written under the names of independent medical researchers […] (Sismondo, 2009: 172).

One might be tempted to suggest that this is just an “a few bad apples” case, but the evidence suggests that publication planning is at play in about 40% of reports of clinical trials on new drugs (Sismondo, 2009: 172). Also, this approach to the publishing process is driven undeniably by commercial interests, particularly by the pharmaceutical industry’s interest of positioning their drugs in the market. For what other reason would pharmaceutical companies spend thousand and even millions of dollars buying reprints of articles and sending them to doctors worldwide, if it did not significantly contribute to profit? As Richard Smith, former editor of *BMJ*, claims:

Finally, companies purchase large numbers of reprints of these trials. Sometimes they will spend more than $lm on reprints of a single study, and the profit margin to the publisher is huge. These reprints are then used to market the drugs to doctors, and the journal’s name on the reprint is a vital part of that sell (Smith, 2003: 1204).

Mainstream academic publishing is thus a major burden for pharmaceutical companies to advertise the relevant research. It is slow and costly. In this sense, open access to scientific publications, would be a great gain for Big Pharma. Even with the gold model, pharmaceutical companies have a huge advantage: they can pay just 3,000 dollars for their article to be published in a well-established medical journal and then distribute it widely without any of the high costs of reprints; certainly, a huge gain.

The epistemic and social problems that arise from publication planning—e.g., the use of authors who did not even contribute to the design and research process, and the conflicts of interest that permeate pharmaceutical research—remain however untouched. In fact, since Open Access policies are only encouraged for publicly funded science, the pharmaceutical companies can open their research to the extent that they find favorable, keeping the publication planning process tightly closed. In other words, they are in a position to take advantage of strategies to open science when they see fit, while maintaining the research process closed when they do not. Furthermore, in this particular case, open access allows pharmaceutical companies to achieve a more efficient publication process, at lesser cost, contributing to strengthening this type of commercialized science.

### Citizen Participation and Citizen Science

An important argument supporting Open Science stems from the idea that science should be more democratic and that the scientific process ought to be open to citizen participation. If we live in a democratic society and science is a key institution for democratic societies to flourish, then scientific projects and scientific results ought to be clearly aligned with society’s needs. This plea for socially responsible science is not constrained to the Open Science movement but has also been a concern of philosophers of science lately (Kitcher, 2001; Kitcher, 2011; Douglas, 2009; Kourany, 2010). In addition, different types of participatory and collaborative methodologies have been developed, especially in the social sciences, in order to include substantive participation of stakeholders in scientific research, where the extent of citizen involvement varies, ranging from mere consent to engaged reciprocity (Wylie, 2015; see also; Koskinen and Mäki, 2016).

The rationale behind these participatory practices is both social and epistemic. On the one hand, participatory research aims to include the views of stakeholders who have been traditionally marginalized in the research process. Opening up the process contributes to increasing the diversity of views, thus reaching better and more reliable knowledge (on the epistemic advantages of diversity see: Longino, 2002 and Harding, 2015). On the other hand, participatory research also aims at social inclusion for those traditionally marginalized in the research process, fostering equality and social justice.

So far, citizen participation within Open Science has been very different from this ideal. Instead of substantive inclusion of stakeholders (their aims and needs), what we have seen is the development of different “citizen science” projects. The name citizen science might be confusing, for it can be understood both as a type of science driven by the concerns and needs of citizens or as scientific projects run by professional scientists, where citizens contribute to data gathering (Elliott, 2019). Let us focus on the latter, the type of citizen science projects that raise the most concern in terms of future commercialization. In these cases, citizen science projects are top-down approaches in which scientists open up the research process selectively, so that citizens can contribute free labor to the project through puzzle-solving or data-gathering. A clear example of this is the Rosetta@home project in which common citizens lend computer processing power while they are not using their devices, to help speed up the effort of protein folding (see https://boinc.bakerlab.org/).

As it turns out, protein folding is incredibly difficult to achieve through mere computer processing, where the computer keeps trying a very large number of possibilities until it finds a proper one. Apparently, the human mind is much faster in coming up with right answers to protein folding problems. For this reason, the Rosetta@home project was rapidly followed by other citizen science projects such as Foldit (see https://fold.it/portal/), a crowdsourcing computer game, where citizens can contribute to finding possible solutions to protein folding using their “human puzzle-solving intuitions.” The website encourages this type of citizen collaboration, claiming that participants will contribute to better understanding disease-related proteins, which could eventually lead to curing diseases, such as HIV, cancer, or Alzheimer’s.

Without critiquing the laudable goals of such projects (no reasonable person would be against finding the cure of mortal diseases and stop human suffering), they are far away from the sort of substantive citizen participation that advocates of democratizing science have in mind. As Powell and Collin claim, “[m]ost participatory exercises do not engage citizens beyond an event or a few weeks/months, and they do not build citizens’ participatory skills in ways that would help them engage with scientists or policy makers independently” (Powell and Collin 2009: 327).

In addition, opening science through citizen science projects is also likely to contribute to further the commercialization of research. Although these projects are for the most part not for profit, run by major research institutions (e.g., University of Washington), in collaboration with government agencies (e.g., The US National Institutes of Health and the US National Science Foundation) (again, publicly-funded research), once relevant results are obtained and potential medications appear in the horizon, the door is open for pharmaceutical companies to buy the results and process the patent. Here again, it seems that the incentives are in place for publicly funded research to do the hard work, now with the help of citizen’s free labor, only for pharmaceutical companies to come in late in the process and profit. As long as universities are able to patent and sell results from government funded research (possible since Bayh-Dole), they have a huge financial incentive to do so, and contribute to the process of commercialization. In this sense, opening research for citizen science projects does not necessarily render socially responsible results.

## Conclusion

In this paper, I have provided a philosophical analysis of Open Science, focusing on the asymmetrical treatment that the Open Science movement gives to public and private research. At first sight, the ideal of Open Science, which promotes the values of transparency, sharing, collaboration, and accountability, seems a promising guideline to address some of the epistemic and social justice problems that have emerged with the rampant commercialization of scientific research. The plea to open science, however, has primarily focused on publicly funded research, leaving research in the private sphere untouched. In fact, advocates of Open Science have used the business opportunities that will potentially emerge from opening publicly funded science as an argument in favor of Open Science, making clear that they are not particularly concerned with the problems of commercialized science. Given that the majority of scientific research is both funded and performed in the private sector today, and that commercially-driven science has important shortcomings that ought to be addressed, the argument of this paper shows that the Open Science movement should seriously consider the way it indirectly supports this commercialization. Taking an explicit stance for opening ALL science, would be more appropriately aligned with the values of transparency, accountability, inclusion, and democracy that the ideal of Open Science endorses.

## Data Availability Statement

The original contributions presented in the study are included in the article/supplementary material, further inquiries can be directed to the corresponding author.

## Author Contributions

As the single author of the paper, MFP is responsible for the paper in its entirety.

## Funding

Funding for this research project was provided by Universidad de los Andes.

## Conflict of Interest

The authors declare that the research was conducted in the absence of any commercial or financial relationships that could be construed as a potential conflict of interest.

## Footnotes

1 I would like to thank one of the anonymous reviewers for pointing out this equivocal use of the concept of open science, as well as the importance of this discussion.

2 The ideal might also emphasize different aspects of the research process depending on the school of thought that promotes it (Fecher and Friesike, 2014).
